# Role of Phosphodiesterase 5 and Cyclic GMP in Hypertension

**DOI:** 10.1007/s11906-016-0646-5

**Published:** 2016-04-14

**Authors:** Evanthia Mergia, Johannes Stegbauer

**Affiliations:** Department of Pharmacology and Toxicology, Ruhr-University Bochum, Bochum, Germany; Department of Nephrology, Medical Faculty, University Hospital Düsseldorf, Heinrich-Heine-University Düsseldorf, Moorenstr. 5, 40225 Düsseldorf, Germany

**Keywords:** cGMP, PDE1, PDE5, Hypertension, Kidney, Sympathetic nerve activity, Natriuresis, Vascular function

## Abstract

Cyclic GMP (cGMP) is a ubiquitous intracellular second messenger that mediates a wide spectrum of physiologic processes in multiple cell types within the cardiovascular and nervous systems. Synthesis of cGMP occurs either by NO-sensitive guanylyl cyclases in response to nitric oxide or by membrane-bound guanylyl cyclases in response to natriuretic peptides and has been shown to regulate blood pressure homeostasis by influencing vascular tone, sympathetic nervous system, and sodium and water handling in the kidney. Several cGMPs degrading phosphodiesterases (PDEs), including PDE1 and PDE5, play an important role in the regulation of cGMP signaling. Recent findings revealed that increased activity of cGMP-hydrolyzing PDEs contribute to the development of hypertension. In this review, we will summarize recent research findings regarding the cGMP/PDE signaling in the vasculature, the central nervous system, and the kidney which are associated with the development and maintenance of hypertension.

## Introduction

Hypertension affects more than 1.5 billion people and is the leading risk factor for cardiovascular morbidity and mortality worldwide [1]. Despite better treatment options and increasing awareness of the fatal consequences of untreated hypertension, control rates for hypertension remain unsatisfactory. Typically, less than 50 % of patients under treatment consistently achieve their blood pressure targets [2]. The reasons for these poor outcomes are complex, but include a relatively limited repertoire of antihypertensive agents and the complex pathophysiology of hypertension involving several physiological key pathways, like the renin-angiotensin-aldosterone system (RAAS), the sympathetic nervous system, the immune system, and the nitric oxide (NO)/cyclic GMP (cGMP) signaling cascade.

While the blockade of the RAAS and the sympathetic nervous system have shown to lower blood pressure in the majority patients, we do not have established any effective treatment option targeting the NO/cGMP signaling cascade for the treatment of hypertension and end organ damage. Here, we will highlight the physiological role of the cGMP generated mainly by NO on blood pressure homeostasis. Moreover, we will focus on the role of the phosphodiesterases which are controlling cGMP availability, and their importance in the development of hypertension.

### Cyclic GMP Signaling

The cGMP signaling cascade is a key regulator in the cardiovascular system controlling vascular tone, water, and salt handling as well as platelet aggregation [3]. Cyclic GMP mediates its effect via different cellular targets: cGMP-dependent protein kinases (cGKs), cGMP-gated cation channels, and phosphodiesterases (PDEs) [4]. For a better understanding how cGMP exerts its effects on blood pressure homeostasis and hypertension, we will first describe the single component of the cGMP signaling cascade in the following section in detail.

## NO-Sensitive Guanylyl Cyclases

Biological signaling by NO is primarily mediated by cGMP. NO generated from l-arginine by different NO synthases binds and activate the heme-containing guanylyl cyclases which in turn converts GTP to the second messenger cGMP. Two isoforms of the NO-sensitive GC exist. The enzymes are heterodimers consisting of a common β_1_ subunit and different α subunits (α_1_, α_2_) and will be here referred to as NO-GC1 (the α_1_β_1_ heterodimer) and NO-GC2 (the α_2_β_1_ heterodimer). Both, NO-GC1 and NO-GC2 have similar sensitivity to NO and to drugs that modulate NO-GC activity [5]. The physiological and pathophysiological roles of the two guanylyl cyclase isoforms are still not fully understood. Analysis of tissue distribution based on measurements of NO-stimulated cGMP formation in NO-GC1 and NO-GC2 knockout mice revealed high levels of NO-GC1 in the lung, kidney, and vascular tissues, whereas comparatively high levels of NO-GC2 occurred in the brain, especially in the hippocampus and medulla oblongata [6–8]. The ability of the C-terminal region of the α_2_ subunit to associate with the PDZ-containing postsynaptic density protein-95 (PSD-95) indicates a synaptosomal localization of the NO-GC2 isoform at least in the neuronal tissues and suggests a possible compartmentalization of the NO/cGMP signaling based on the localization of the cGMP-forming isoform [9]. In platelets, NO-GC1 was the sole NO-GC responsible for the NO-mediated inhibition of platelets aggregation [10].

## Membrane-Bound Receptor Guanylyl Cyclases

In addition to NO, the natriuretic peptides can also increase intracellular cGMP levels by activation of membrane-bound guanylyl cyclase receptors. Of the seven membrane-bound GCs, the guanylyl cyclase A (GC-A) is mainly involved in blood pressure regulation and hypertension [11, 12]. The GC-A is activated by the atrial natriuretic peptide (ANP) and the brain natriuretic peptide (BNP), two primarily cardiac hormones which are secreted from the atrium and ventricle during pressure or volume overload. Increases of cGMP levels induced by ANP or BNP mediates physiological effects such as vascular relaxation, modulation of endothelial permeability, inhibition of renin, and aldosterone secretion, as well as salt and water handling in the kidney. Thus, recent studies have shown that inhibition of ANP and BNP degradation by a neprilysin inhibitor combined with an angiotensin II receptor blocker improves heart failure and resistant hypertension in human and mice [13–15].

## The Phosphodiesterases: Key Regulator of cGMP Bioavailability

Beside the GCs, the cyclic nucleotide PDEs are controlling cGMP signaling as these enzymes regulate the breakdown of cGMP into its inactive form, GMP. PDEs comprise a diverse family of enzymes: 11 different families, each consisting of one to four isoforms and multiple splice variants [16]. Of the 11 member group PDE5, PDE6, and PDE9 are highly selective for cGMP, PDE1, PDE2, and PDE11 have dual-specificity (cAMP and cGMP), and PDE3 and PDE10 are cGMP-sensitive but cAMP-selective. Interestingly, any particular cell type might express three or four different PDEs. For example, in vascular smooth muscle cells, three different PDEs are responsible for cGMP degradation: PDE1, PDE3, and PDE5 [17]. The expression of different PDEs in the same cell seem to be of major regulatory significance as they are responsible for compartmentalization of the cGMP signaling which translate specific extracellular cues in selective activation of downstream targets. Therefore, it is not surprising that the different PDEs are activated under different conditions. In this regard, the PDE1 is only fully active after binding of Ca2+ and calmodulin. Thus, it has been shown that increase of intracellular Ca2+ levels induced by various potent vasoconstrictors, such as norepinephrine, angiotensin II, and endothelin-1 induce PDE1 activation which in turn serves to lower cGMP levels and augment vasoconstriction even further [17].

**Fig. 1:**
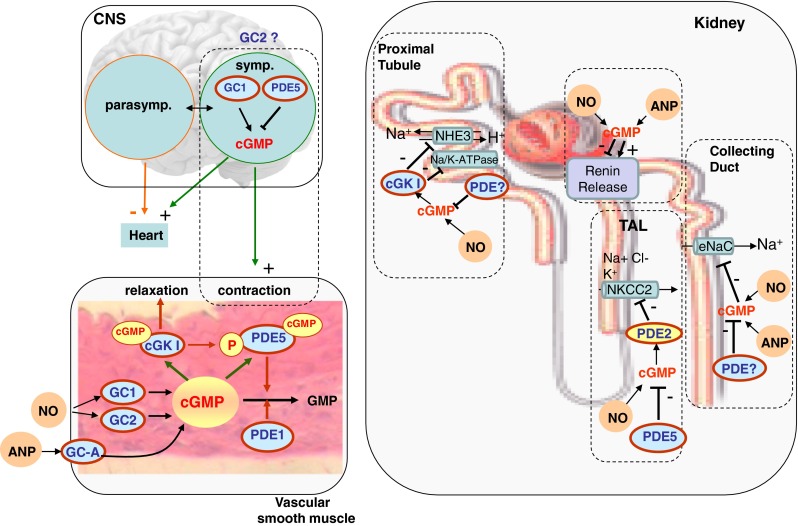
Schematic overview of cGMP signaling in vascular smooth muscle cells, the central nervous system (CNS), and the renal nephron. In the vascular smooth muscle cells, cGMP can be formed by the NO-sensitive guanylyl cyclases (GC1 and GC2) or the membrane-bound guanylyl cyclase A (GC-A) in response to NO or ANP, respectively. Increased cGMP levels lead to activation of cGMP-dependent protein kinase I (cGK I) and relaxation. Parallel cGMP causes allosteric activation of PDE5 which is accompanied by cGK I-mediated phosphorylation. Cyclic GMP-dependent PDE5 activation acts as negative feedback that limits the cGMP response. Beside PDE5, also PDE1 participates to the hydrolysis of cGMP. Additional to vascular signaling, cGMP plays a role in the central nervous system (CNS). In the medulla oblongata, a sympatho-stimulatory action of cGMP formed by the GC1 has been proposed. Increased sympathetic output is also reported using the PDE5 inhibitor sildenafil. GC2 is also expressed in the medulla oblongata, but its role remains elusive. In the renal nephron cGMP signaling initiated either by NO or ANP has a modulatory role on the renin secretion. Moreover cGMP increases natriuresis (1) by reducing surface expression of the type 3 Na+/H+ exchanger (NHE3) and the Na+/K + −ATPase in the proximal tubular cells, (2) by inhibiting trafficking of Na-K-2Cl co-transporter (NKCC2) in the thick ascending limb (TAL), and (3) by inhibiting the epithelial Na + channel (eNaC) in the collecting duct

On the other hand, the PDE5 activity is increased under conditions of increased cGMP generation. In detail, PDE5 is activated by allosteric binding of cGMP to the tandem regulatory GAF domains, so named from the first three classes of proteins found to contain the cGMP-binding sequence (mammalian cGMP-binding PDEs, Anabaena adenylate cyclase, and Escherichia coli Fh1A) (Abbildung 1). Binding of cGMP to the PDE5 GAF domain increases catalytic affinity and catalytic rate of the enzyme. Parallel to activation of cGMP production, PDE5 can be phosphorylated by the cGMP-dependent protein kinase (cGK I) in its N-terminal region. Phosphorylation prolongs PDE5 activation most likely by increasing the affinity toward cGMP but, even without phosphorylation, PDE5 activation is much sustained [18, 19]. This negative feedback mechanism has been proposed to regulate the sensitivity of the NO/cGMP signaling and has been shown to be responsible for the transient shape of the cGMP response (rapid cGMP increase and breakdown) in platelets [18]. Further this mechanism is also sufficient to explain the NO-induced desensitization, i.e., the reduced cGMP accumulation following a second stimulation with NO [20, 21]. The close interaction of PDE5 with the NO/cGMP signaling is also well documented by the fact that the effectiveness of PDE5 inhibitors depends on the cGMP forming capacity. Pharmacologic inhibition of NOS, which abrogates NO synthesis and subsequent cGMP formation, suppresses the vasodilator effects of PDE5 inhibition. Thus PDE5 inhibitors are expected to be less effective in disorders associated with reduced NOS activity, such as endothelial dysfunction. Conversely, PDE5 inhibitors (e.g., sildenafil) are able to greatly potentiate the effects of NO generating compounds. Accordingly, although sildenafil had only a modest effect on reduction of systemic blood pressure, this could turn into severe hypotension for patients taking a combination of sildenafil and nitroglycerin or other organic nitrates [22].

### PDE5 and cGMP Signaling in The Vasculature

Since the discovery of NO, the role of the NO/cGMP signaling cascade regulating vascular tone has been demonstrated in many studies [23]. An impairment of this pathway results in vascular dysfunction and hypertension [20, 24]. Beside a decreased NO bioavailability, increased activity of cGMP-degrading PDEs in vascular smooth muscle cells is the main cause for vascular dysfunction characterized by exaggerated vasoconstrictor response and impaired NO-dependent vasodilation. Vascular dysfunction alters vascular tone and contributes to development of hypertension. Until now, the circumstances leading to an activation of either PDE1 or PDE5 are still not known. In vascular smooth muscle cells (VSMC), augmentation of intracellular Ca^2+^ levels induced by angiotensin II have been shown to increase PDE1 expression [25]. In line with this, PDE1-dependent reduction of cGMP availability has been shown to contribute to the increased vasoconstrictor response observed in chronic angiotensin II infusion [26]. Interestingly, PDE1 more than PDE5 seem to play also an important role in age-dependent vascular dysfunction. In mice with defective nucleotide excision repair gene (Ercc1d/−), a mouse model which develops severe age-related vascular dysfunction, inhibition of both PDE1 and PDE5 restored vascular dysfunction. In addition, senescent human VSMC have elevated PDE1A, PDE1C and PDE5 mRNA levels. Moreover, a single nucleotide polymorphism in PDE1A was associated with elevated diastolic blood pressure and vascular hypertrophy suggesting a role of the PDE1A isoform in human vascular function [27•]. On the other side, recent studies have shown a higher contribution of PDE5 to vascular dysfunction observed in experimental hypertension [7, 28–30]. In renovascular hypertension, an initially increased NO/cGMP signaling leads to cGMP-dependent PDE5 activation (Fig. 1) which in turn enhances cGMP degradation and promotes vascular dysfunction [7]. In support of this concept, inhibition of the PDE5 by sildenafil increased renal cGMP content, restored renal and mesenteric vascular function, and reduced blood pressure in renovascular hypertension [7, 28, 29]. Beside the direct effects of PDE5 inhibition on vascular function, sildenafil also seems to exert its effect on blood pressure by reducing angiotensin II levels and restoring the baroreflex in renovascular hypertension [28, 29].

### PDE5 and NO/cGMP Signaling in The Central Nervous System

Increasing evidence from device-based therapeutic interventions to decrease renal and systemic nerve activity in patients with resistant hypertension points to an essential role of the sympathetic nervous system in the etiology of hypertension [21].

The activity of the sympathetic nerves that regulate cardiovascular function due to their effects to increase cardiac rate and contractility, cause vasoconstriction, release of adrenal catecholamines, and activate the RAAS is determined by a network of neurons located in the medulla oblongata. Here, a controversial role of NO in the synaptic transmission that regulates sympathetic output has been proposed. For example, local application of NO donors in the nucleus tractus solitarii (NTS) has been shown to elicit inhibitory or excitatory effects on sympathetic nerve activity [31–33]. Likewise, administration of NO into the rostral ventrolateral medulla (RVLM) also demonstrates inhibitory or excitatory effects on sympathetic nerve activity in rats [34–36].

In the medulla, both NO-GCs that serve as receptor for NO are expressed [8]. Localization of the NO-GCs on different points of the neural network in the medulla may help us to explain the discrepant findings of the NO signaling in controlling sympathetic nerve activity. Initial evidence suggesting separate roles and cellular distribution of the two NO-GC isoforms in the synaptic transmission came from studies in the hippocampus using KO mice deficient of NO-GC1 or NO-GC2. These studies demonstrated that the NO-GC1 is located presynaptically at both glutamatergic and GABAergic synapses and involved in the regulation of neurotransmitter release [37, 38]. In contrast therefore, the NO-GC2 is located postsynaptically at glutamatergic synapses and responsible for enhancing postsynaptic responsiveness [39•]. Promising results suggesting a more directed role for NO-GC1 in the regulation of sympathetic activity and blood pressure were shown in a recent study [40]. NO-GC1 KO mice on a C57Bl/6 background are not hypertensive, despite reduced vascular relaxation and increased vascular tone. This was found to be most likely related to a decreased sympathetic nerve activity in NO-GC1 KO mice, as plasma norepinephrine levels and heart rates were lower compared to WT mice. In line with this finding, blood pressure response to chronic norepinephrine but not to chronic angiotensin II infusion was exaggerated in NO-GC1 KO than in WT mice [40].

Whether PDE5 also regulates NO/cGMP signaling in neurons as in vascular smooth muscles cells is not widely studied. Compared to a ubiquitous expression of PDE5 in vascular smooth muscle cells of different vascular beds, PDE5 expression in the brain is more regional restricted. Human PDE5 mRNA was found to be the highest in the cerebellum, medulla, and spinal cord [41]. Yet, there are no specific data which provide evidence of PDE5 expression in brain areas controlling cardiovascular effects, but several physiologic studies suggest a direct central effect of PDE5 inhibition by sildenafil. For example, there are reports that sildenafil induces an increase in muscle sympathetic nerve activity in healthy volunteers as well as an increase in plasma catecholamine levels [42, 43]. Sildenafil injected into the central nervous system (lateral cerebral ventricles) of rats increased lumbar sympathetic nerve activity and caused tachycardia without changes in arterial pressure [44]. Recently, Dopp et al. reported that sildenafil increases sympathetically mediated vascular tone and plasma norepinephrine concentrations in middle-aged men [45]. These observations suggest that sildenafil in contrast to its direct vaso-protective and blood pressure lowering effects in the vasculature enhances sympathetic mediated vasoconstriction. This sympathetic activation may limit the blood pressure effect of sildenafil and may explain why PDE5 inhibitors co-administered with alpha-blockers induce a pronounced systemic vasodilation and severe hypotension [46]. Although it could be questioned whether sildenafil is only targeting PDE5 or also PDE1, which is more highly expressed in the brain, the overall data presented above support the view that cGMP signaling beside to its vascular effect also exerts a direct central sympathetic effect [47].

On the other hand, different clinical studies reported that sildenafil reduces blood pressure, which was characterized by a slight or no effect on heart rate suggesting that sildenafil does not increase sympathetic activity in humans [48, 49]. In this context, a new study suggests that PDE5 inhibition by sildenafil reduces blood pressure in the setting of renovascular hypertension by restoring baroreflex sensitivity [28]. In sum, further studies are needed to determine the overall effect of NO/cGMP-PDE5 signaling in central nervous system on blood pressure regulation and hypertension.

### Cyclic GMP Signaling in The Kidney

Beside the sympathetic nervous system and the peripheral vasculature, the kidneys exert an outstanding role in blood pressure homeostasis and hypertension by regulating salt and water handling as well as vascular tone. One of the key mechanisms leading to the hypertension is an activated renin-angiotensin system and defective pressure natriuresis. The cGMP signaling pathway is able to modulate renin secretion, pressure natriuresis, and renal vascular resistance.

## Renin Release

Depending on yet still unknown mechanisms, cGMP generated either by NO or ANP is able to stimulate or inhibit renin release in vitro and vivo. Under most circumstances, cGMP generated by NO stimulates the renin release through a PDE3-dependent inhibition of cAMP degradation [50]. In addition, recruitment of renin expressing cells along the preglomerular vessels is strongly dependent on the NO/cGMP signaling cascade [51]. On the other side, Kurtz and colleagues have also demonstrated that cGMP by activating the cGMP-dependent kinase II can also inhibit renin release [52].

## Cyclic GMP Signaling Influences Natriuresis and Hypertension

In the past few years, various studies highlighted the effect of cGMP on fluid and salt transport within the nephron. Under hypertensive blood pressure conditions, cGMP signaling plays an important role in regulating pressure natriuresis by affecting the solute transport of several sodium channels in the proximal tubule, the thick ascending limb, and the collecting duct like the Na^+^/K^+^-ATPase, the type 3 Na^+^/H^+^ exchanger (NHE3), the Na^+^/K^+^/2Cl^−^ cotransport (NKCC), and the epithelial sodium channel (eNaC), respectively [53, 54, 55••, 56, 57]. In the proximal tubule, cGMP has been shown to increase phosphorylation of Src-kinase and initiate thereby signaling events that are capable of reducing the surface expression of apical NHE3 and basolateral Na^+^/K^+^-ATPase leading to increased pressure natriuresis [58, 59•]. The relevance of cGMP signaling in regulating pressure natriuresis was confirmed in a recent study showing that a polymorphism in the cGMP-dependent protein kinase I was associated with salt-sensitive hypertension and impaired pressure natriuresis after salt load in humans. These cGMP-related polymorphisms resulted in a loss of pressure natriuresis control in a Src-Na^+^/K^+^-ATPase dependent manner [60]. In line with these human data, Kemp and colleagues have shown that selective activation of the angiotensin II type 2 (AT2) receptor induced the internalization/inactivation of Na^+^/K^+^-ATPase and NHE3 in proximal tubule leading to pressure natriuresis and attenuated blood pressure response in experimental hypertension. Again, these effects seemed to be mediated by a NO/cGMP-dependent mechanism which involved the signaling kinases ERK1/2 and Src [61••]. However, it should be noted that a recent finding indicate that the effect of the NO/cGMP signaling cascade on proximal tubule transport seemed to be species dependent and dependent on the circumstances how cGMP is generated. Thus, in contrast to the results seen in mice, Shirai et al. could demonstrate that angiotensin II activates NHE3, and the basolateral Na + −HCO_3_^−^ co-transporter through a NO/cGMP-dependent mechanism in human proximal tubular cells [62•].

Until now, the responsible phosphodiesterase which is controlling cGMP availability and thereby proximal tubular transport is not known. However, recent data have shown that chronic PDE5 inhibition increased expression of cGMP-dependent protein kinase I in proximal tubule cells and thereby attenuated the development of renal fibrosis in a mouse model of unilateral urethral obstruction [63].

Similar to the proximal tubule, cGMP signaling regulates blood pressure by influencing sodium and water transport in the distal tubule [64]. In the thick ascending limb, cGMP exerts its natriuretic effect by inhibiting cAMP-mediated trafficking of Na-K-2Cl co-transporter (NKCC2) through the activation of the cGMP-stimulated PDE2 which lowers cAMP levels [65]. Beside PDE2, recent data examining the effect of NO on NKCC2 function suggest that PDE5 activation reduces cGMP availability and thereby increases sodium reabsorption via the NKCC2 in angiotensin II-dependent hypertension (Fig. 1). In addition, inhibition of PDE5 increases cGMP levels and restores the inhibitory effect of NO on NKCC2 in the thick ascending limb [55••]. The source of NO mediating this effect can be of quite different origins. Thus, it has been shown that luminal flow-induced shear stress stimulates NO generation and thereby inhibits NKCC2 [66]. In addition, a recent study revealed a very interesting finding on macrophages modulating the extent of hypertension. In interleukin1 receptor (IL-1R)-deficient mice, angiotenin II induced hypertension was attenuated compared to wild type mice. This effect was explained by kidney infiltrating macrophages of IL-1R-deficient mice that produce increased NO and thereby inhibit NKCC2 which results in increased natriuresis [67••].

In the collecting duct, the final segment of the distal tubule where the composition of the urine can be regulated, cGMP generated in response to either ANP or NO mediates its natriuretic effects by inhibiting the epithelial Na + channel (eNaC) [56, 57]. Recently, Hyndman and colleagues have shown that deletion of the neuronal NO synthase (NOS1) within the collecting duct result in salt-sensitive hypertension and reduced urine output [68]. Studies in pregnant rat revealed that the natriuretic effect caused by cGMP signaling in the collecting duct is attenuated by increased PDE5 activity. Inhibition of the PDE5 by sildenafil restored natriuresis in these rats [69].

## Conclusion

The pathogenesis of hypertension is a multifactorial process. Decrease of cGMP signaling in the blood vessels, in the central nervous system, or in the kidney contributes to the development and maintenance of hypertension. Among the causes that decreased cGMP availability, enhanced degradation by PDE5 plays a central role. Thus, PDE5 inhibition leads to blood pressure reduction via vasodilation and increased pressure natriuresis by affecting several sodium transporters within the kidney. Moreover, first clinical trials suggest that PDE5 inhibition not only reduces blood pressure but also protects from chronic kidney disease [70]. However, the potential of PDE5 inhibition as a promising therapeutic option in the treatment of hypertension has to be tested in large clinical trials.
